# Metformin as a cellular protector; a synoptic view of modern evidences

**Published:** 2015-01-01

**Authors:** Nicolas Wiernsperger

**Affiliations:** ^1^INSERM U1060, CarMeN Laboratory, INSA Lyon, Claude Bernard University, Villeurbanne, France

**Keywords:** Metformin, Lactic acidosis, Apoptosis, Cell

## Abstract

Due to limited knowledge and chemical class effect assimilation the biguanide metformin has long been considered as a useful but risky treatment for type 2 diabetes treatment. The worldwide long-term experience of clinical use of this compound and the growing knowledge about its mechanisms of action have, however, reversed this reputation to the point that nowadays it is not only considered as relatively harmless but even increasingly as a cellular protector. The present mini-review simply aims at giving a brief overview of the evidences accumulated overt recent periods and to provide the reader with information as to mechanistic hypotheses, knowing that there remains a lot to be done to better understand the pleiotropic behavior of this drug and its possible future new therapeutic applications. Data are shown at a glance for the kidney but also for other various organs and cell types corroborating this new notion for an old drug and paradox.

**Implication for health policy/practice/research/medical education:**

The worldwide long-term experience of clinical use of metformin and the growing knowledge about its mechanisms of action have, however, reversed this reputation to the point that nowadays it is not only considered as relatively harmless but even increasingly as a cellular protector.

## Introduction

Since its launching in 1957 metformin has been the subject of several thousands of preclinical and clinical reports which have finally evidenced its pleiotropic properties, largely contrasting with its clinical use essentially limited to first line type 2 diabetes therapy until today. However, looking back to this broad literature, this was not unexpected: in the early 70’s one could already find clear-cut reports showing that biguanides in general (of which almost only metformin has survived) exert many different effects on cellular systems (1). Without doubt, the main reason for this monolithic application as the fear of lactic acidosis, which has indeed been found in patients with renal insufficiency (although mainly using phenformin and buformin). Due to favorable circumstances metformin could fortunately escape the ban of this chemical class, although it was for long time considered to be a dangerous drug. It is only after 3-4 decades that its long-term, world-wide clinical use raised some concerns about reality of life-threatening drug effects in patients with low renal clearance and, since such situations were accompanied by high plasma drug levels, the correlation was likely established between those two parameters. Here and now, however, some casual reports suggest that, in contrast to the common belief, sometimes patients may have survived thanks to the drug (2). Clearly this is not denying the occurrence of fatal cases of lactic acidosis with metformin, rather it raises concerns about the incidence of the claimed direct relationship. Just recently it was proposed that some patients might be hyposensitive toward metformin, in contrast to other, hypersensitive ones (3).

The present review, therefore, will present recent accumulating data showing that indeed metformin can protect cells from death in various cell types and pathological situations, some of which appear quite spectacular.

## Metformin and the kidney

In view of the long-lasting debate about renal insufficiency and possible metformin harmfulness, we will start with this topic. Here, however, we will only briefly summarize the actual state of knowledge since excellent reviews on this subject have recently been published in this journal (4) as well as in a series of papers by Lalau (5,6).

### 1. Renal insufficiency/lactic acidosis

When renal clearance is hampered, drug elimination is decreased, leading to its plasma and organ accumulation. Various pharmacokinetic investigations as well as case reports of patients with lactic acidosis suggested that metformin builds up a so-called “reservoir” which, although not firmly established, is located in the intestinal wall and possibly in erythrocytes. Although metformin does not increase lactate production in the latter cell type (no mitochondria present) it amounts to concentrations reaching 10 mM in some layer of the intestinal wall (7). Lactate is easily produced by intestine (one of its physiological roles) and cleared by the portal vein to reach to liver and then the blood stream. Liver, as well as other organs such as the heart, is one main organ clearing lactate from blood, notably using it as a precursor of gluconeogenesis. Hepatocytes are equipped with lactate transporters and some pathological situations do interfere with the liver’s capacity to normally clear lactate from the portal vein, one of which is a reduction in hepatic blood flow which may happen in shock situations. In addition metformin at high concentrations such as measured in the portal vein can inhibit lactate transport by the hepatocytes. It is therefore likely that in severe clinical conditions hepatic lactate clearance will be hampered by the simultaneous operation of both the above mentioned factors. This was indeed verified in experimental conditions (8) and in clinical dossiers of lactic acidosis cases (Wiernsperger, unpublished observations).

In his extensive analysis of many reported cases of lactic acidosis seemingly associated with metformin, Lalau came to the conclusion that renal insufficiency was generally not harmful for patients, a conclusion also reached in recent meta-analysis (9). Other analyses suggest that various critical factors can lead to lactic acidosis even in the absence of chronic renal failure (10). We suggest therefore that mainly the combination of renal and other organ failures, notably hepatic insufficiency, may lead to possibly lethal lactic acid levels. Even then, however, the presence of metformin is not necessarily linked to fatal outcome since even rescues attributed to metformin have been claimed (2). The various mechanisms reported for cellular protection by metformin (see later) fully support this notion.

### 2. Renal cell protection by metformin

Data on possible protection of kidney function by metformin are logically scarce since the drug was claimed to be nephrotoxic. However, recent advances showed that metformin is indeed able to protect the kidney from various injuries, as is reported in other organs. In vivo metformin improves the biochemical changes generated by diabetes in rat kidneys (11); in cultured mouse podocytes the drug reduced NAD (P)H oxidase activity, increased AMPK and extracellular ATP and finally stimulated P2 purinergic receptors (12). AMPK activation was reported to protect proximal tubular cells submitted to various forms of stress-induced apoptosis; this occurred together with stimulation for the Akt pathway, while ATP changes were not causally involved (13). Chronic treatment of diabetic rats with metformin, although only mildly reducing hyperglycemia, opposed to the renal lesions in particular the podocyte loss (14). Here again the protective drug effect was attributed to inhibition of oxidative stress. Pre-activation of AMPK by metformin also improved post-ischemic recovery of the kidney epithelial cells (15).

In tubular epithelial cells, but not in mesangial cells, metformin inhibited the unfolded protein response in response to glucosamine and deoxyglucose (16). The effect appeared independent of AMPK activation, similar to the protection seen in human renal proximal tubular epithelial cells (17); in the latter experiment metformin appeared to reduce the hypoxia-inducible factor (HIF-1) accumulation.

The data show that even without a strong reduction of diabetic hyperglycemia, metformin has direct protective effects on various kidney cells, corroborating in vitro data in renal cultured cells. The mechanisms whereby the drug positively affects these cells appear to involve primarily inhibition of oxidative stress, activation of AMPK being observed but not necessarily involved.

## Metformin and cardiovascular system

Vascular protective effects of metformin are known since decades but were largely described in the past two decades. Many studies have demonstrated improvements in cellular and hemostatic parameters, both in diabetic and non-diabetic situations (18), thereby confirming the pleiotropic nature of this compound (19). In preclinical investigations the vasculo-protective drug effects were seen in doses generally well below those used to combat hyperglycemia. One should particularly mention unique effects of metformin on the microcirculation such as stimulation of arteriolar vasomotion, inhibition of permeability and leucocyte adhesion, all features explaining the remarkable post-ischemic improvement of microvascular blood flow permitting conservation of tissue integrity (20). The positive effects on microcirculation were also considered to mainly explain the better outcome of metformin-treated diabetic patients in the long-term UKPDS study (21). Interestingly the microvascular effects of metformin have been confirmed in human subjects presenting various degrees of prediabetic, normoglycemic conditions (22).

Improvements in limb circulation were described long time ago in patients with peripheral vascular diseases (23). More recently data provided demonstrations in the cardiology field: a very low dose, preventive administration of metformin remarkably reduced myocardial infarction in rodents (24) confirming data obtained in rabbits (25). Troponin 1 and creatine kinase were reduced after coronary intervention and 1 year later the frequency of cardiovascular events was 8% vs 28% (26). Chronic heart failure (27) and myocardial infarction (28) were reportedly improved by metformin. Another study reported no reduction in number of cardiovascular events but improved survival (29).

## Metformin and cell death in various systems

In various cell types and under various experimental conditions metformin was shown to inhibit apoptosis. In conditions close to diabetes, metformin reduced programmed cell death in endothelial cells under hyperglycemic conditions (30-32), but also in the presence of advanced glycation endproducts in Schwann cells (33). The protection was as good as with cyclosporine A (30).

The inhibition of apoptosis by metformin has been described in many cell types and under most various conditions. Reduced cell death and ceramide synthesis as well as increased AMPK activity were reported in cardiomyocytes in presence of palmitic acid with metformin concentrations below 5 mM (34). Similar observations were made in hepatocytes added with bile acids (35) or saturated fatty acids (36), in neurons stressed by etoposide (37), in glial cells (38), in pancreatic islet with free fatty acids (39,40). Metformin inhibited apoptosis in auditory cells with gentamicine or cisplatin (41,42), and this property received confirmation in vivo in irradiated guinea-pigs (43).

## Metformin and senescence

It should be mentioned here that in recent years several (but not all) reports mentioned that metformin had life-prolonging effects similar to caloric restriction (44) in primitive worms and some rat strains (45-48). Mechanistic investigations suggest that inhibition of mTOR may be responsible since this appears to be a critical target in aging (49). These data are still preliminary but look promising.

## Metformin and cancer cell apoptosis

Based on newly discovered mechanisms of metformin action, its potential benefit in cancer has generated plenty of publications over the past years. Many encouraging data are reported, although the clinical results still await confirmation (50). Interestingly metformin, in contrast to non-cancer cells, seems to be capable of pro-apoptotic behavior in cancer cells: positive reports exist for a number of different cancer cell types, for example breast cells (51), renal A498 cells (52), esophageal squamous cells (53), hepatoma cells (54) and others (55). High, pharmacological doses of metformin can also induce apoptosis in normal cells (34,56-58). Thus caution should be given to the interpretation of experimental in vitro data mostly using supra-normal irrelevant metformin concentrations.

## Mechanisms of metformin cellular protection

Not surprisingly when considering the pleiotropic effects of metformin, many different mechanisms to explain the protection afforded by metformin have been proposed. Although not the aim of this article, some of which are listed below for reader’s information. Two main ones are reduction of oxidative stress and stimulation of AMPK. Metformin has been shown to reduce oxidative stress in many experimental and clinical conditions. One mechanism for reducing oxidative stress is a partial inhibition of mitochondrial complex I (59-61) and inhibition of PTP opening (62,63). While repeatedly found, however, this mechanism has not been confirmed in human diabetics (64). Moreover other measurements have also shown that mitochondrial energy production was increased by metformin (65,66). AMPK activation has frequently been claimed to be responsible for the large diversity of biological effects reported with metformin. However there are many reports of increased AMPK activity by metformin without direct causal involvement in the observed effects. Clearly AMPK activation is not the sole mechanism of action of metformin (67). Stimulation of the PI3-Akt pathway seems also important (35). Here again, the experimental conditions (model, drug dose) must be considered as a key determinant of obtained results, a characteristic which is reflected in the often apparently paradoxical results published such as seen for AMPK, PARP or apoptosis as such.

Among many other mechanisms involved (but not necessarily causally), reduction of the endoplasmic reticulum stress has been claimed to be a target of metformin but this hypothesis needs more evidence (68). Diminution of glycation, either by direct quenching (69,70) or reduction of RAGE receptors (71) can partly explain improvements under hyperglycemic conditions.

## Conclusion

In sharp contrast with the ancient belief that biguanides were toxic, including a limited toxicity for the specific drug metformin, the clinical experience and the biocellular researches performed over the last two decades have provided a complete “renaissance” of this antidiabetic compound. Indeed, the literature now weekly issues new publications showing demonstration or strong evidence for multiple protective effects of metformin, the most suggestive – but still to be proved – being some forms of cancer. This is typical for so-called pleiotropic compounds and for metformin the likely explanation is a stabilization of membrane structure and fluidity (72). In its traditional clinical application in diabetes studies have shown that the metformin-associated risk of lactic acidosis is clearly far below what was thought, although not denying its existence. Comorbidities, however, seem generally involved in these few cases. In recent years suggestions even appear to lessen the restrictions applied to metformin use (73,74). Growing evidence is found for positive effects of metformin in the cardiovascular area, notably chronic heart failure and myocardial infarction. These human data are to be expected on the basis of the unique vascular pharmacological properties of the drug. However, looking at the direct cellular level it is recognized that metformin protects various cell types against death (in particular apoptosis) induced by various insults. On the contrary it looks like it might favor apoptosis in cancer cells. A lot is clearly still to be found with this old but still largely ignored compound.

## Footnote

The literature about metformin is extremely abundant (several thousand articles). The present article is conceived as a mini-review, accordingly the bibliography is to be looked at as a minimal referring to main notions about the topic of the paper.

## Author’s contribution

NW was the single author of the paper.

## Conflict of interests

The author declared no competing interests.

## Ethical considerations

Ethical issues (including plagiarism, misconduct, data fabrication, falsification, double publication or submission, redundancy) have been completely observed by the author.

## Funding/Support

None

## References

[1] Schäfer G (1976). On the mechanism of action of hypoglycemia-producing biguanides A reevaluation and a molecular theory. Biochem Pharmacol.

[2] Kajbaf F, Arnouts P, de Broe M, Lalau JD (2013). Metformin therapy and kidney disease: a review of guidelines and proposals for metformin withdrawal around the world. Pharmacoepidemiol Drug Saf.

[3] Lalau JD, Azzoug ML, Kajbaf F, Briet C, Desailloud R (2014). Metformin accumulation without hyperlactataemia and metformin-induced hyperlactataemia without metformin accumulation. Diabetes Metab.

[4] Nasri H, Baradaran A, Ardalan MR, Mardani S, Momeni A, Rafieian-Kopaei M (2013). Bright renoprotective properties of metformin: beyond blood glucose regulatory effects. Iran J Kidney Dis.

[5] Lalau JD, Race JM (2001). Lactic acidosis in metformin therapy: searching for a link with metformin in reports of ‘metformin-associated lactic acidosis’. Diabetes Obes Metab.

[6] Lalau JD, Arnouts P, Sharif A, De Broe ME (2014 Mar 5). Metformin and other antidiabetic agents in renal failure patients. Kidney Int.

[7] Wilcock C, Bailey CJ (1994). Accumulation of metformin by tissues of the normal and diabetic mouse. Xenobiotica.

[8] Barthelmebs M, Wiernsperger N, Krieger JP, Rapin JR, Radziuk J, Grima M (2003). Mild acute renal failure potentiates metformin accumulation in the diabetic rat kidney without further impairment of renal function. Diabetes Metab.

[9] Salpeter SR, Greyber E, Pasternak GA, Salpeter EE (2010). Risk of fatal and nonfatal lactic acidosis with metformin use in type 2 diabetes mellitus. Cochrane Database Syst Rev.

[10] Rocha A, Almeida M, Santos J, Carvalho A (2013). Metformin in patients with chronic kidney disease: strengths and weaknesses. J Nephrol.

[11] Alhaider AA, Korashy HM, Sayed-Ahmed MM, Mobark M, Kfoury H, Mansour MA (2011). Metformin attenuates streptozotocin-induced diabetic nephropathy in rats through modulation of oxidative stress genes expression. Chem Biol Interact.

[12] Piwkowska A, Rogacka D, Jankowski M, Angielski S (2013). Metformin reduces NAD(P)H oxidase activity in mouse cultured podocytes through purinergic dependent mechanism by increasing extracellular ATP concentration. Acta Biochim Pol.

[13] Lieberthal W, Zhang L, Patel VA, Levine JS (2011). AMPK protects proximal tubular cells from stress-induced apoptosis by an ATP-independent mechanism: potential role of Akt activation. Am J Physiol Renal Physiol.

[14] Kim J, Shon E, Kim CS, Kim JS (2012). Renal podocyte injury in a rat model of type 2 diabetes is prevented by metformin. Exp Diabetes Res.

[15] Seo-Mayer PW, Thulin G, Zhang L, Alves DS, Ardito T, Kashgarian M (2011). Preactivation of AMPK by metformin may ameliorate the epithelial cell damage caused by renal ischemia. Am J Physiol Renal Physiol.

[16] Thériault JR, Palmer HJ, Pittman DD (2011). Inhibition of the Unfolded Protein Response by metformin in renal proximal tubular epithelial cells. Biochem Biophys Res Commun.

[17] Takiyama Y, Harumi T, Watanabe J, Fujita Y, Honjo J, Shimizu N (2011). Tubular injury in a rat model of type 2 diabetes is prevented by metformin: a possible role of HIF-1α expression and oxygen metabolism. Diabetes.

[18] Wiernsperger NF (2000). Metformin: intrinsic vasculoprotective properties. Diabetes Technol Ther.

[19] Schäfer G (1983). Biguanides A review of history, pharmacodynamics and therapy. Diabete Metab.

[20] Wiernsperger NF, Bouskela E (2003). Microcirculation in insulin resistance and diabetes: more than just a complication. Diabetes Metab.

[21] Turner RC (1998). Turner RCThe UKProspective Diabetes StudyA review. Diabetes Care.

[22] Kraemer de Aguiar LG, Laflor CM, Bahia L, Villela NR, Wiernsperger N, Bottino DA (2007). Metformin improves skin capillary reactivity in normoglycaemic subjects with the metabolic syndrome. Diabet Med.

[23] Sirtori CR, Franceschini G, Gianfranceschi G, Sirtori M, Montanari G, Bosisio E (1984). Metformin improves peripheral vascular flow in nonhyperlipidemic patients with arterial disease. J Cardiovasc Pharmacol.

[24] Calvert JW, Gundewar S, Jha S, Greer JJ, Bestermann WH, Tian R (2008). Acute metformin therapy confers cardioprotection against myocardial infarction via AMPK-eNOS-mediated signaling. Diabetes.

[25] Wiernsperger N, Rapin JR: unpublished results.

[26] Li J, Xu JP, Zhao XZ, Sun XJ, Xu ZW, Song SJ (2014). Protective effect of metformin on myocardial injury in metabolic syndrome patients following percutaneous coronary intervention. Cardiology.

[27] Ekeruo IA, Solhpour A, Taegtmeyer H (2013). Metformin in diabetic patients with heart failure: safe and effective?. Curr Cardiovasc Risk Rep.

[28] Whittington HJ, Hall AR, McLaughlin CP, Hausenloy DJ, Yellon DM, Mocanu MM (2013). Chronic metformin associated cardioprotection against infarction: not just a glucose lowering phenomenon. Cardiovasc Drugs Ther.

[29] Lamanna C, Monami M, Marchionni N, Mannucci E (2011). Effect of metformin on cardiovascular events and mortality: a meta-analysis of randomized clinical trials. Diabetes Obes Metab.

[30] Detaille D, Guigas B, Chauvin C, Batandier C, Fontaine E, Wiernsperger N (2005). Metformin prevents high-glucose-induced endothelial cell death through a mitochondrial permeability transition-dependent process. Diabetes.

[31] Arunachalam G, Samuel SM, Marei I, Ding H, Triggle CR (2014). Metformin modulates hyperglycaemia-induced endothelial senescence and apoptosis through SIRT1. Br J Pharmacol.

[32] Mahrouf-Yorgov M, Marie N, Borderie D, Djelidi R, Bonnefont-Rousselot D, Legrand A (2009). Metformin suppresses high glucose-induced poly(adenosine diphosphate-ribose) polymerase overactivation in aortic endothelial cells. Metabolism.

[33] Ota K, Nakamura J, Li W, Kozakae M, Watarai A, Nakamura N (2007). Metformin prevents methylglyoxal-induced apoptosis of mouse Schwann cells. Biochem Biophys Res Commun.

[34] An D, Kewalramani G, Chan JK, Qi D, Ghosh S, Pulinilkunnil T (2006). Metformin influences cardiomyocyte cell death by pathways that are dependent and independent of caspase-3. Diabetologia.

[35] Woudenberg-Vrenken TE, 
Conde de la Rosa
 
L
, Buist-Homan M, Faber KN, Moshage H (2013). Metformin protects rat hepatocytes against bile acid-induced apoptosis. PLoS One.

[36] Kim J, Shon E, Kim CS, Kim JS (2012). Renal podocyte injury in a rat model of type 2 diabetes is prevented by metformin. Exp Diabetes Res.

[37] El-Mir MY, Detaille D, R-Villanueva G, Delgado-Esteban M, Guigas B, Attia S (2008). Neuroprotective role of antidiabetic drug metformin against apoptotic cell death in primary cortical neurons. J Mol Neurosci.

[38] Liu X, Chhipa RR, Pooya S, Wortman M, Yachyshin S, Chow LM (2014). Discrete mechanisms of mTOR and cell cycle regulation by AMPK agonists independent of AMPK. Proc Natl Acad Sci U S A.

[39] Lupi R, Del Guerra S, Fierabracci V, Marselli L, Novelli M, Patanè G (2002). Lipotoxicity in human Pancreatic islets and the protective effect of metformin. Diabetes.

[40] Simon-Szabó L, Kokas M, Mandl J, Kéri G, Csala M (2014). Metformin Attenuates Palmitate-Induced Endoplasmic Reticulum Stress, Serine Phosphorylation of IRS-1 and Apoptosis in Rat Insulinoma Cells. PLoS One.

[41] Chang J, Jung HH, Yang JY, Choi J, Im GJ (2011). Protective role of antidiabetic drug metformin against gentamicin induced apoptosis in auditory cell line. Hear Res.

[42] Chang J, Jung HH, Yang JY, Lee S, Choi J, Im GJ (2014). Protective effect of metformin against cisplatin-induced ototoxicity in an auditory cell line. J Assoc Res Otolaryngol.

[43] Mujica-Mota MA, Salehi P, Devic S, Daniel SJ (2014). Safety and otoprotection of metformin in radiation-induced sensorineural hearing loss in the guinea pig. Otolaryngol Head Neck Surg.

[44] Dhahbi JM, Mote PL, Fahy GM, Spindler SR (2005). Identification of potential caloric restriction mimetics by microarray profiling. Physiol Genomics.

[45] Onken B, Driscoll M (2010). Metformin induces a dietary restriction-like state and the oxidative stress response to extend C elegans Healthspan via AMPK, LKB1, and SKN-1. PLoS One.

[46] Anisimov VN, Berstein LM, Egormin PA, Piskunova TS, Popovich IG, Zabezhinski MA (2008). Metformin slows down aging and extends life span of female SHR mice. Cell Cycle.

[47] Cabreiro F, Au C, Leung KY, Vergara-Irigaray N, Cochemé HM, Noori T (2013). Metformin retards aging in C elegans by altering microbial folate and methionine metabolism. Cell.

[48] Martin-Montalvo A, Mercken EM, Mitchell SJ, Palacios HH, Mote PL, Scheibye-Knudsen M (2013). Metformin improves healthspan and lifespan in mice. Nat Commun.

[49] Lamming DW, Ye L, Sabatini DM, Baur JA (2013). Rapalogs and mTOR inhibitors as anti-aging therapeutics. J Clin Invest.

[50] Micic D, Cvijovic G, Trajkovic V, Duntas LH, Polovina S (2011). Metformin: its emerging role in oncology. Hormones (Athens).

[51] Queiroz EA, Puukila S, Eichler R, Sampaio SC, Forsyth HL, Lees SJ (2014). Metformin Induces Apoptosis and Cell Cycle Arrest Mediated by Oxidative Stress, AMPK and FOXO3a in MCF-7 Breast Cancer Cells. PLoS One.

[52] Fang Z, Xu X, Zhou Z, Xu Z, Liu Z (2014). Effect of metformin on apoptosis, cell cycle arrest migration and invasion of A498 cells. Mol Med Rep.

[53] Feng Y, Ke C, Tang Q, Dong H, Zheng X, Lin W (2014). Metformin promotes autophagy and apoptosis in esophageal squamous cell carcinoma by downregulating Stat3 signaling. Cell Death Dis.

[54] Saito T, Chiba T, Yuki K, Zen Y, Oshima M, Koide S (2013). Metformin, a diabetes drug, eliminates tumor-initiating hepatocellular carcinoma cells. PLoS One.

[55] Luo Q, Hu D, Hu S, Yan M, Sun Z, Chen F (2012). In vitro and in vivo anti-tumor effect of metformin as a novel therapeutic agent in human oral squamous cell carcinoma. BMC Cancer.

[56] Kefas BA, Cai Y, Kerckhofs K, Ling Z, Martens G, Heimberg H (2004). Metformin-induced stimulation of AMP-activated protein kinase in beta-cells impairs their glucose responsiveness and can lead to apoptosis. Biochem Pharmacol.

[57] Yi G, He Z, Zhou X, Xian L, Yuan T, Jia X (2013). Low concentration of metformin induces a p53-dependent senescence in hepatoma cells via activation of the AMPK pathway. Int J Oncol.

[58] Leclerc GM, Leclerc GJ, Kuznetsov JN, DeSalvo J, Barredo JC (2013). Metformin induces apoptosis through AMPK-dependent inhibition of UPR signaling in ALL lymphoblasts. PLoS One.

[59] Batandier C, Guigas B, Detaille D, El-Mir MY, Fontaine E, Rigoulet M (2006). The ROS production induced by a reverse-electron flux at respiratory-chain complex 1 is hampered by metformin. J Bioenerg Biomembr.

[60] Masini M, Anello M, Bugliani M, Marselli L, Filipponi F, Boggi U (2014). Prevention by metformin of alterations induced by chronic exposure to high glucose in human islet beta cells is associated with preserved ATP/ADP ratio. Diabetes Res Clin Pract.

[61] Wheaton WW, Weinberg SE, Hamanaka RB, Soberanes S, Sullivan LB, Anso E (2014). Metformin Inhibits mitochondrial complex I of cancer cells to reduce tumorigenesis. Elife.

[62] Leverve XM, Guigas B, Detaille D, Batandier C, Koceir EA, Chauvin C (2003). Mitochondrial metabolism and type-2 diabetes: a specific target of metformin. Diabetes Metab.

[63] Guigas B, Detaille D, Chauvin C, Batandier C, De Oliveira F, Fontaine E (2004). Metformin inhibits mitochondrial permeability transition and cell death: a pharmacological in vitro study. Biochem J.

[64] Larsen S, Rabøl R, Hansen CN, Madsbad S, Helge JW, Dela F (2012). Metformin-treated patients with type 2 diabetes have normal mitochondrial complex I respiration. Diabetologia.

[65] Vytla VS, Ochs RS (2013). Metformin increases mitochondrial energy formation in L6 muscle cell cultures. J Biol Chem.

[66] Kristensen JM, Larsen S, Helge JW, Dela F, Wojtaszewski JF (2010). Two weeks of metformin treatment enhances mitochondrial respiration in skeletal muscle of AMPK kinase Miller RA, Birnbaum MJ An energetic tale of AMPK-independent effects of metformin. J Clin Invest.

[67] Miller RA, Birnbaum MJ (2010). An energetic tale of AMPK-independent effects of metformin. J Clin Invest.

[68] Cai H, Zhang G, Chen W, Zhang B, Zhang J, Chang J (2014). Metformin protects the myocardium against isoproterenol-induced injury in rats through alleviating endoplasmic reticulum stress. Pharmazie.

[69] Ruggiero-Lopez D, Lecomte M, Moinet G, Patereau G, Lagarde M, Wiernsperger N (1999). Reaction of metformin with dicarbonyl compounds Possible implication in the inhibition of advanced glycation end product formation. Biochem Pharmacol.

[70] Beisswenger PJ, Howell SK, Touchette AD, Lal S, Szwergold BS (1999). Metformin reduces systemic methylglyoxal levels in type 2 diabetes. Diabetes.

[71] Zhang T, Hu X, Cai Y, Yi B, Wen Z (2014). Metformin protects against hyperglycemia-induced cardiomyocytes injury by inhibiting the expressions of receptor for advanced glycation end products and high mobility group box 1 protein. Mol Biol Rep.

[72] Wiernsperger NF (1999). Membrane physiology as a basis for the cellular effects of metformin in insulin resistance and diabetes. Diabetes Metab.

[73] Adam WR, O’Brien RC. A justification for less restrictive guidelines on the use of metformin in stable chronic renal failure. Diabet Med 2014 Jun 9. 10.1111/dme.12515 24909998

[74] Juurlink DN, Roberts DM (2014). The enigma of metformin-associated lactic acidosis. Clin Toxicol (Phila).

